# Cardiac output measurement in Malawian children ages 2 months–12 years hospitalised with severe anaemia (COM-TRACT)

**DOI:** 10.1093/inthealth/ihaf103

**Published:** 2026-07-03

**Authors:** Elizabeth Chintolo, Roisin Connon, Elizabeth C. George, George Chagaluka, Bridon M’baya, A. Sarah Walker, Neil Kennedy, Kathryn Maitland

**Affiliations:** a College of Medicine, Malawi-Liverpool-Wellcome Trust Clinical Research Programme, PO Box 30096, Chichiri, Blantyre 3, Malawi; b Medical Research Council Clinical Trials Unit at University College, 90 High Holborn London WC1V 6LJ, UK; c Malawi Blood Transfusion Services, PO Box 2681Blantyre, Malawi; d School of Medicine, Dentistry and Biomedical Science, Queen’s University, 97 Lisburn Road, Belfast BT97BL UK; e Department of Infectious Disease and Institute of Global Health and Innovation, Division of Medicine, Imperial College, Praed Street, London W2 1PG UK

**Keywords:** echocardiography, Malawian children, severe anaemia transfusion, USCOM

## Abstract

**Background:**

Little is known about myocardial perturbations in African children hospitalised with severe anaemia.

**Methods:**

An observational study nested within a clinical trial of blood transfusion was conducted on the paediatric ward in Blantyre, Malawi. Children were ages 2 months–12 years hospitalized with uncomplicated severe anaemia (haemoglobin 4–6 g/dl). By randomisation, 13 children received 30 ml/kg whole blood, 13 received 20 ml/kg whole blood and 26 had no immediate transfusion (usual care). We measured standard parameters of cardiac function using ultrasonic cardiac output monitoring (USCOM) at enrolment, 8 and 24 hours and discharge.

**Results:**

Fifty-two children, median age 39 months (interquartile range [IQR] 25–58) and median haemoglobin 5.1 g/dl (IQR 4.8–5.6) were studied. Severe tachycardia and tachypnoea over time corrected faster in the transfused arms than the controls. At enrolment, the stroke volume index was within the normal range and 26/52 (50%) had a cardiac output index (COI) > 97.5% the standard centile. The COI decreased in all arms by discharge but was greatest in the transfusion arms (p = 0.05 for 20 ml/kg and p = 0.009 for 30 ml/kg). A higher volume or receipt of whole blood did not worsen cardiac function. No child required diuretics.

**Conclusions:**

The data generated by this small but granular study of haemodynamic and cardiac function provide reassuring physiological evidence showing the safety of higher doses of blood transfusion than currently recommended. It also supports the findings of a secondary analysis of the Transfusion and Treatment of Severe Anaemia in African Children trial indicating that whole blood transfusions are safe. These data support the new evidence-based paediatric transfusion algorithm for anaemic African children and its recommendation for safe use.

## Introduction

The Global Burden of Disease 2019 study estimated that anaemia affects 30% of children, with some of the highest national point prevalences occurring in sub-Saharan Africa (SSA). In various settings, severe anaemia (defined as a haemoglobin [Hb] <6 g/dl) is a key cause of paediatric hospital admission (present in 6–22% of admissions)^1, 2^ and results in a high demand for blood for emergency transfusion. The aetiology of severe anaemia in African children is frequently multifactorial^3^ but largely secondary to infectious causes (malaria and sepsis), nutritional deficiencies^4^ and sickle cell anaemia.^5^ In SSA the only comprehensive case–control study of children hospitalised with severe anaemia, conducted in Blantyre, Malawi,^3^ demonstrated the key associations with severe anaemia were bacteraemia (odds ratio [OR] 5.3 [95% confidence interval {CI} 2.6 to 10.9]), malaria (OR 2.3 [95% CI 1.6 to 3.3]), hookworm infestation (OR 4.8 [95% CI 2.0 to 11.8]), human immunodeficiency virus (HIV) infection (OR 2.0 [95% CI 1.0 to 3.8]), vitamin A deficiency (OR 2.8 [95% CI 1.3 to 5.8]) and vitamin B12 deficiency (OR 2.2 [95% CI 1.4 to 3.6]). Neither iron nor folate deficiencies were associated with mortality and were less prevalent among cases than hospital or community controls. In the Transfusion and Treatment of Severe Anaemia in African Children Trial (TRACT) involving Ugandan and Malawian children hospitalised with severe anaemia, baseline characteristics indicated that 63% had *Plasmodium falciparum* malaria and 22% had sickle cell disease (by genotype) while HIV infection, culture-proven bacteraemia and severe malnutrition were all uncommon (<4%).^6^

Transfusion of blood can be a life-saving intervention, and provision of adequate supplies of safe blood for transfusion are an essential undertaking for any health system. The pattern of usage of blood in SSA is very different from that of high-income countries, as use is largely elective with supply strictly monitored through specialist transfusion services. In SSA, children < 5 y of age account for 54% of blood transfusions issued, most given as emergency interventions.^7, 8^ Owing to high demand and limited transfusion resources, the World Health Organization developed guidelines for the appropriate use of blood for patient groups suffering the greatest impact from a shortage of supply.^9^ For children, the guidelines encourage restrictive transfusion approaches, specifically not transfusing stable children with Hb of 4–6 g/dl.^10^ Transfusion is recommended for children with profound anaemia (Hb <4 g/dl) and for children with Hb of 4–6 g/dl with additional severity features (including respiratory distress, altered consciousness and shock).

Thus children hospitalised with severe anaemia, considered for immediate transfusion, frequently have signs compatible with compensatory hyperdynamic circulation. These include tachycardia, a bounding pulse and a systolic flow murmur.^11^ Nevertheless, these signs overlap with the definitions of cardiac failure/overload, leading to current recommendations to cautiously transfuse and to give this with an accompanying diuretic.^10^ This guidance remains deeply ingrained in the clinical management of children hospitalised with severe anaemia, even though it is based on a weak evidence base and has been superseded by new evidence.^6, 12^ The multicentre phase III TRACT demonstrated that both increased transfusion volume in afebrile children, compared with standard volumes recommended,^12^ and whole blood, compared with red cell concentrates,^13^ lead to better outcomes. Given the current guideline recommendations, willingness to adopt the new consensus transfusion management algorithm^14^ based on the evidence provided by the TRACT may prove challenging.

The TRACT^7^ provided an opportunity to explore in a nested substudy of haemodynamic and myocardial perturbations and changes over time in children with uncomplicated severe anaemia who did or did not receive blood. In addition, we looked at whether a higher volume of blood (30 ml/kg whole blood equivalent) compared with the recommended standard volume (20 ml/kg whole blood equivalent) was safe. We hypothesised that the data obtained may provide reassuring physiological evidence of the safety of the treatment strategies and help to support revisions to the World Health Organization (WHO) paediatric transfusion guidelines for resource-limited settings.

The primary objectives of this substudy were to determine cardiac output (CO), stroke volume (SV), cardiac output index (COI) and stroke volume index (SVI) in children presenting to the hospital with uncomplicated severe anaemia and to determine the response of cardiac output to transfusion or non-transfusion.

## Methods

### Study design and participants

The TRACT^7^ was an open-label, multicentre, factorial randomized trial involving 3983 hospitalised children in three centres in Uganda (Mulago National Referral Hospital [in Kampala], Mbale Regional Referral Hospital and Soroti Regional Referral Hospital) and one centre in Malawi (Queen Elizabeth Central Hospital, Blantyre), enrolling children ages 2 months–12 y admitted with severe anaemia (Hb <6 g/dl). The sites were selected to represent a diversity of malaria transmission. In eastern Uganda, malaria endemicity is high and perennial (Mbale and Soroti centres), in Kampala (Mulago) malaria transmission is low and in Blantyre, in southern Malawi, malaria endemicity in moderate and highly seasonal. The details of trial management and conduct have been published previously.^15^ Cardiac output monitoring in the TRACT (COM-TRACT) was an exploratory observational substudy restricted to children enrolled in the Queen Elizabeth Central Hospital in the TRACT B stratum. This stratum included children ages 2 months–12 y hospitalized with uncomplicated severe anaemia (Hb 4–6 g/dl) with no alterations of consciousness level, respiratory distress (defined as an increased work of breathing), acute haemoglobinuria^16^ or disclosed sickle cell disease.^6^ Children with known chronic disease (renal/liver failure, malignancies, congenital heart disease) or admitted for burns, trauma or surgery were also excluded, as were children with previous transfusions during the same admission.

### Measurements and procedures

Children with suspected severe anaemia (i.e. severe pallor) were assessed for eligibility by the dedicated trial team. Hb was measured using the HemoCue Hb 301 system (HemoCue, Angelholm, Sweden) and a structured clinical assessment was performed. Following enrolment, children were randomised 2:1:1 to no immediate transfusion (standard of care), immediate transfusion with 30 ml/kg of whole blood (15 ml/kg red cell concentrate^17^) or 20 ml/kg whole blood (10 ml/kg red cell concentrate^17^). A baseline and subsequent assessments were recorded on dedicated case report forms by the trial clinicians. In addition, bedside observations were performed at admission and every 30 min for the first 2 h, then 4, 8, 16, 24 and 48 h after the start of the first transfusion by the study nurses and recorded on trial-specific observation charts. Hb was assessed using the HemoCue Hb 301 system every 8 h in the first 24 h, then at 48 h or if triggered by clinical deterioration.^18^ At each bedside review participants were actively monitored for serious adverse events, particularly suspected cardiac or pulmonary overload or transfusion-related events.^19^ In the controls (no immediate transfusion arm), children were eligible for a 20 mg/kg whole blood equivalent transfusion if they developed severe and complicated anaemia (Hb < 4 g/dl and/or development of severity symptoms [increased work of breathing and or alteration of consciousness level]). Other treatments, including antimalarial and antibiotic agents, were administered according to national guidelines.

### CO monitoring

In the COM-TRACT, CO was measured at the Malawi site using ultrasonic cardiac output monitoring (USCOM; USCOM, Coffs Harbour, NSW, Australia), which provides rapid, non-invasive measurement of haemodynamic parameters including CO. A recent meta-analysis comparing USCOM measurements of CO with gold-standard pulmonary artery thermodilution during cardiac catheterisation concluded that USCOM is ‘the first choice for non-invasive echocardiography for the measurement of CO’. USCOM uses continuous-wave Doppler ultrasound to measure the velocity of blood flow through the aortic and pulmonary valves via a probe placed on the suprasternal notch on the chest.^20^ Once the optimal flow profile is obtained, the trace is frozen on the screen and CO is automatically calculated (a product of the heart rate and SV). The SV represents the product of the velocity–time integral and the cross-sectional area of the chosen valve based on the patient’s weight and height. Normal paediatric values, according to age, of peak aortic flow measured with suprasternal ultrasound have been previously published by Cattermole et al.^21^

USCOM measurements were made at four time points—at/soon after enrolment (T0), i.e. prior to any transfusion (in those randomised to immediate transfusion); 8 h post-transfusion (T8); 24 h post-transfusion (T24) and before discharge from the hospital (TD)—along with bedside measurements of heart rate, blood pressure and other clinical variables. Measurements were made by a clinician who was independent of the clinical trial team. The average of three Doppler wave measurements, observed over 10–15 min, were taken as the cardiac function variables per time point. Real-time data on SV, CO, SVI and COI were generated. However, USCOM measurements were not shared with the trial teams and therefore not used to guide clinical decision-making.

### Sample size, data management and statistical analysis

For the main trial, the statistician at the Medical Research Council Clinical Trials Unit at University College London generated and kept the sequential randomization list, computer-generated using variably sized permuted blocks. Data entry was conducted in duplicate, at each site, onto an online password-protected open-source database (OpenClinica) and data management was overseen by the data manager at Kenya Medical Research Institute–Wellcome Trust Research Programme, Kilifi, Kenya, in conjunction with the trial statisticians in London.

For this COM-TRACT study, a convenience sample was used, as there is no reference evidence to support and power the sample size. Basic descriptive statistics were used to describe the distribution of bedside vital signs and USCOM variables. *χ*^2^ and Kruskal–Wallis tests were used to compare categorical and continuous data. Means and 95% CIs were plotted over time for bedside vital signs, Hb and lactate measures. SVI and COI were also plotted over time and then analysed with linear regression, adjusted for the baseline value to estimate the change by arm at discharge. Data were analysed after completion of the main TRACT (2019) by intention-to-treat analysis.

## Results

**Figure 1:**
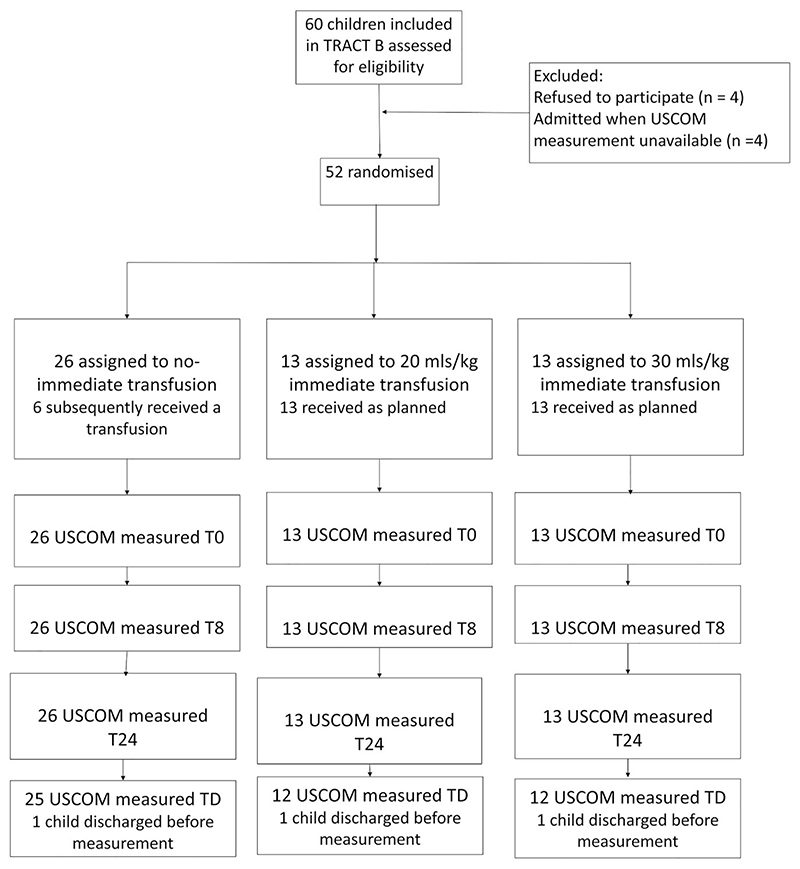
CONSORT diagram.

Between 1 February and 31 August 2016, 52 participants enrolled in the TRACT were included in the COM-TRACT substudy (Figure 1). Baseline characteristics are presented in Table 1. The median age was 39 months (IQR 25–58), slightly older than those enrolled in TRACT B in the main trial (26 months [IQR 12–50]). The median Hb was 5.1 g/dl (IQR 4.8–5.6) with no differences between study arms. Key comorbidities included malaria (40 [76.9%]) and HIV (5 [9.6%]). Both undernutrition and sickle cell anaemia were uncommon. With respect to other severity indices (that did not meet the WHO definition of severity indices for severely anaemic children), age-adjusted severe tachycardia and age-adjusted severe tachypnoea were present in 14 (26.9%) and 30 (57.7%) patients, respectively; clinical signs of compensated shock were present in 22 (43.1%) patients and lactate > 2 mmol/l were present in 37 (78.7%) patients. By design, none had an altered consciousness level or increased work of breathing, as these were exclusion criteria for the TRACT B stratum. All children randomised to immediate 20 ml/kg or 30 ml/kg received their transfusion; 6 of 26 children in the control group had a deferred transfusion (5/6 received it > 48 h after admission, 1/6 received it 26 h after admission) due to the development of a clinical severity sign (consciousness level or increased work of breathing) or a decrease in Hb to <4 g/dl. Of those in the COM-TRACT substudy receiving blood transfusions, 81.2% (26/32) were given as whole blood. As described in the main trial report, no participants developed a transfusion-associated cardiac overload (TACO) or transfusion-related acute lung injury (TRALI). No child was prescribed or required diuretics during the trial.^6^

### Bedside vital signs, Hb and lactate changes

**Figure 2:**
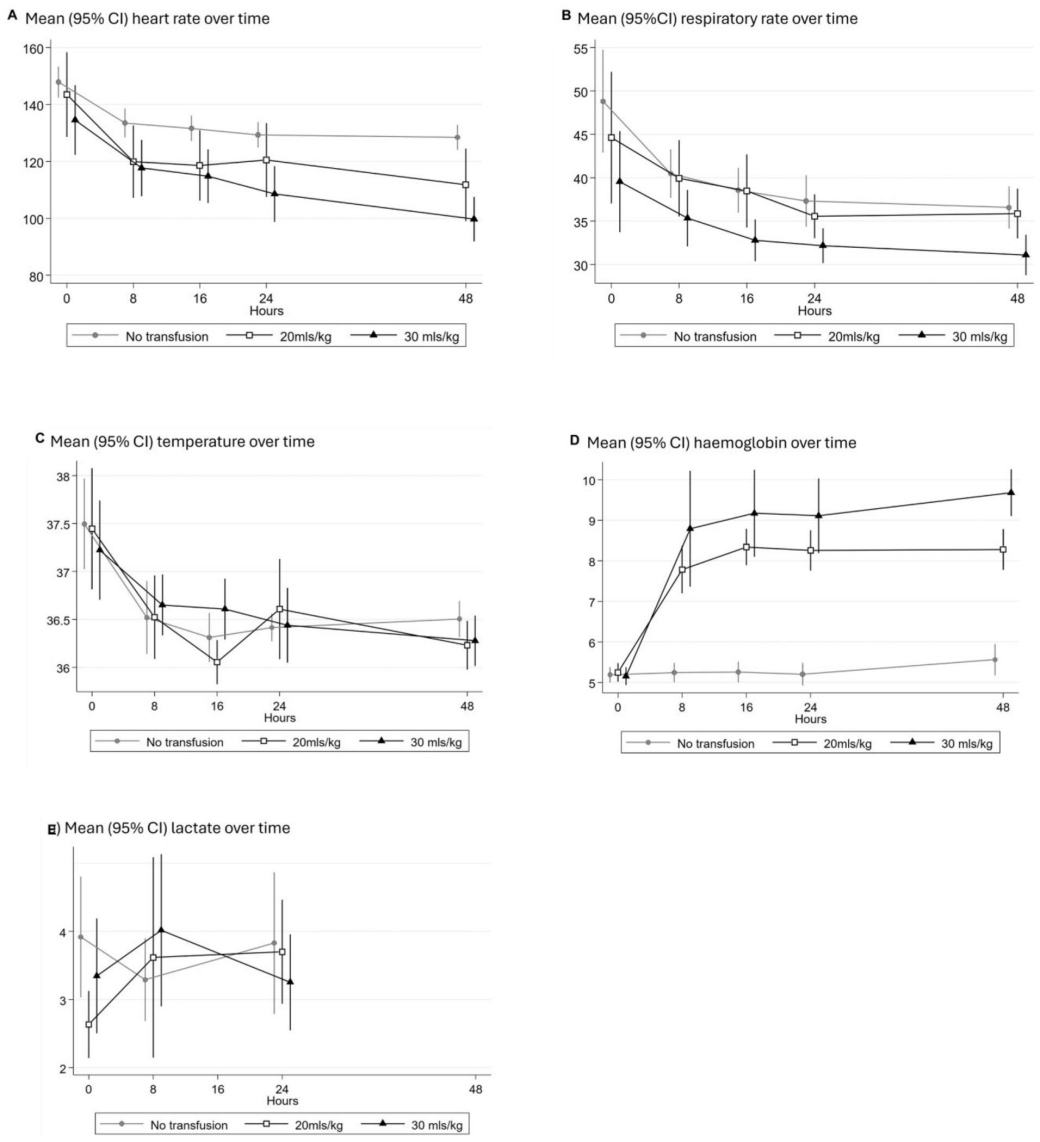
Changes in vital signs and haemodynamics over time.

The heart rate, respiratory rate and temperature decreased in all groups, with the largest decrease seen early in admission (Figure 2a–c). Hb levels were all similar at the time of randomisation across the groups. As expected, by 8 h Hb levels in the intervention arms had risen substantially, with the greatest increase in children receiving 30 ml/kg; with no change in the control arm (Figure 2d). By 24 and 48 h, mean Hb levels remained significantly higher in both the 20 ml/kg (p < 0.001) and 30 ml/kg (p < 0.001) arms compared with controls. During the first 24 h of admission, lactate levels remained similar in each arm.

### CO parameters

As a result of the children randomised to 30 ml//kg being slightly older (and heavier), there were significant differences at baseline between the groups for SV and CO (Table 1). However, we did not observe differences between the groups for the SVI and COI measures at baseline. As both SVI and COI take account of the body surface area of participants, further analysis was therefore restricted to SVI and COI.

**Figure 3:**
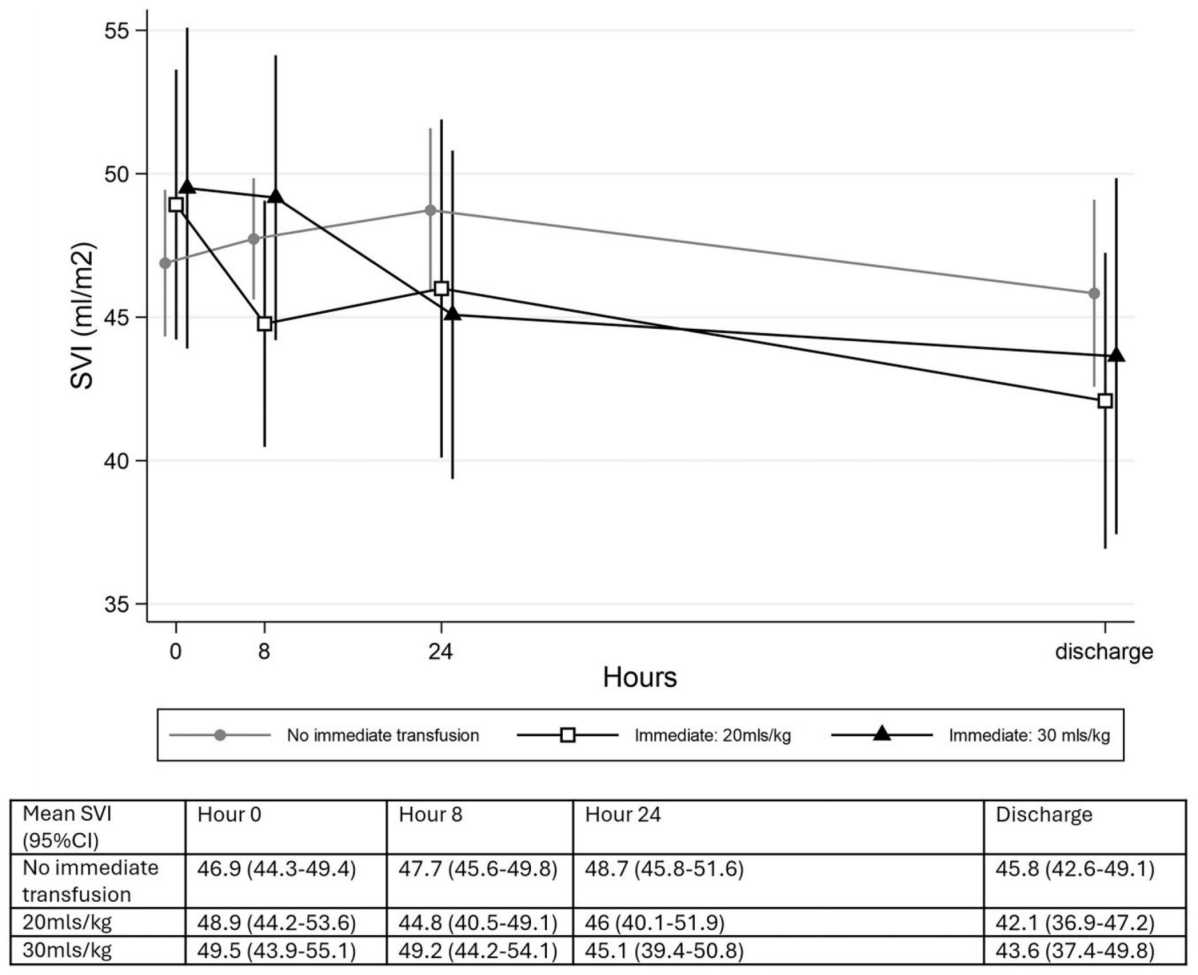
SVI over time.

SVI at baseline was within the normal range of published values measured using USCOM for all except one participant, who had a value > 97.5%. In the control group, SVI (Figure 2) did not change over the time period (−1.3% [95% CI −5.1 to 2.5]), but SVI decreased in both transfusion arms (20 ml/kg: −5.5% [95% CI −11.2 to 0.9], p = 0.05); 30 ml/kg: −7.3% [95% CI −12.7 to −1.9], p = 0.009] (Figure 3). There was no evidence for a difference in the rate of decrease between arms (p = 0.17).

**Figure 4:**
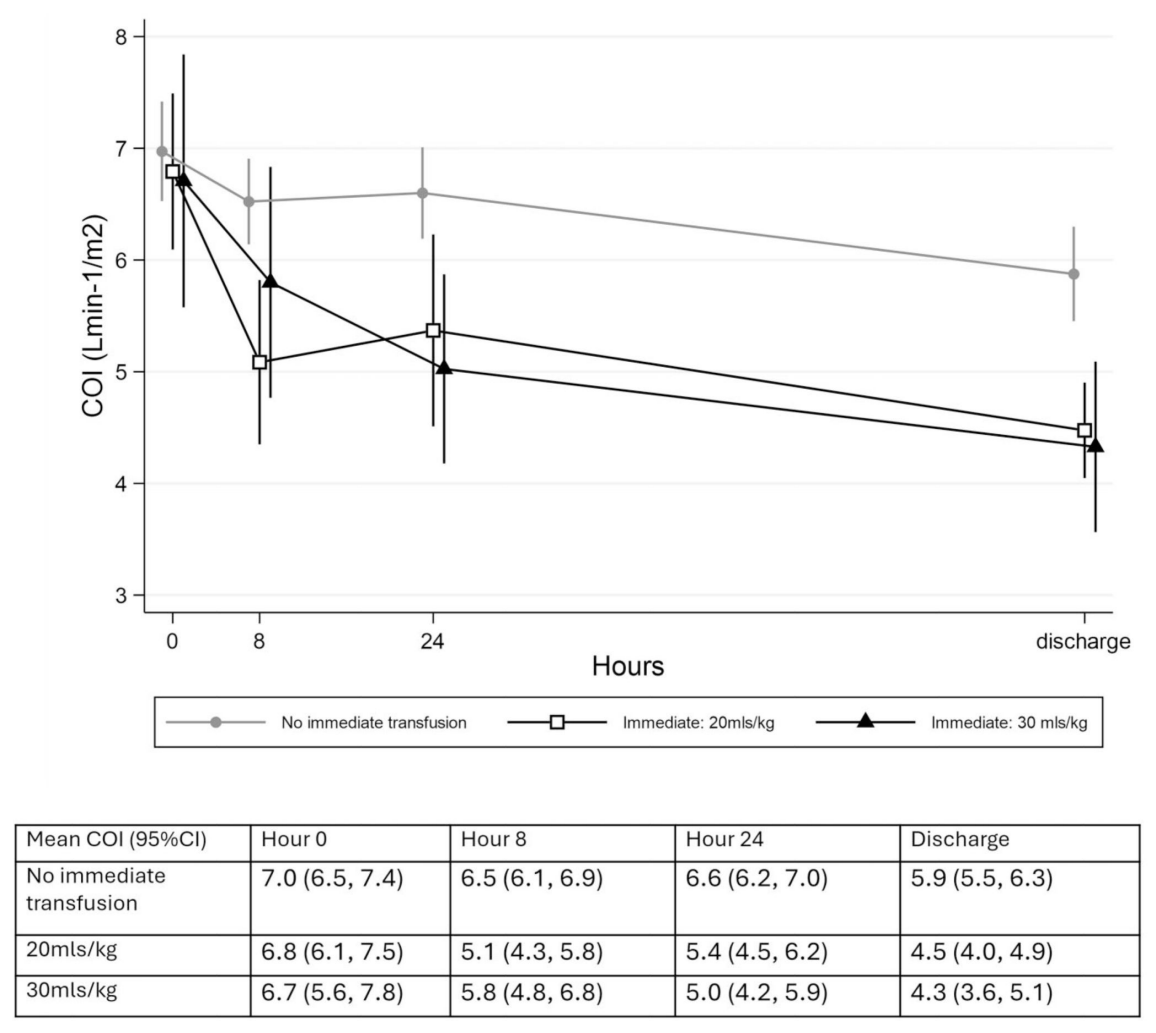
COI over time.

In contrast to SVI, COI (Figure 4) at baseline was > 97.5% of published values^21^ for 26 of 52 (50%) participants. After baseline, COI decreased in all arms. Most of this decrease occurred in the first 24 h of admission. Only six participants had a value >97.5% on discharge; four of these were in the control group. Compared with the controls (reduction of −1.1% [95% CI − 1.5 to −0.7] over time), the COI decreased significantly more quickly in both the 20 ml/kg (−2.5% [95% CI −3.1 to −2.0], p<0.001) and 30 ml/kg (−2.4% [95% CI −3.0 to −1.9), p<0.001] arms. There was no difference in the rate of decrease between the transfusion groups (p = 0.80). Supplementary Tables S1 and S2 show the regression models for unadjusted change in SVI at 24 h and unadjusted and adjusted COI change at 24 h, respectively.

## Discussion

In this study of 52 Malawian children presenting to hospital with severe anaemia, the majority had signs of compensatory hyperdynamic circulation at presentation. COIs were high in all participants at admission/enrolment. However, the rate of COI reduction was significantly higher among children receiving an immediate transfusion than in children in the control (no immediate transfusion) group. The reduction in COI seen in all participants was mediated mainly by a reduction in heart rate, and to a lesser extent by a reduction in SV. Although SVI decreased between admission and discharge in children receiving an immediate transfusion, it remained relatively constant in children in the control group. Reassuringly the SVIs of children included in this study were within the published normal range for nearly all participants. We found no evidence of worsening of any of the parameters in all arms (including control), which is reassuring and supports the main findings of the TRACT.^6, 12^

Our findings are similar to those of a small study of children with severe uncomplicated anaemia due to renal failure or iron deficiency^28^ that reported a significant decrease in COI following transfusion. A study of 185 children with malaria in Ghana reported a decrease in both COI and SVI between admission and recovery 6 weeks later (mean COI 5.8 l/min/m^2^ [standard deviation {SD} 1.8] vs 4.7 [SD 1.4]; mean SVI 40.8 ml/m^2^ [SD 10.5] vs 29.1 [SD 7.6]).^22^ The values reported for both COI and SVI were lower in the Ghanian study than in our study. This can largely be explained by differences in measurement technique (USCOM vs estimation of SV from transthoracic echocardiography), morbidity (only 40% of our participants had malaria) and Hb levels (7.4 g/dl vs 5.1 in our study). It is known that infants increase CO mainly by increasing their heart rate; in contrast, older children and adolescent do so mainly by increasing SV.^29^ In our study, most participants were younger (median age 39 months), thus it is not surprising that a decrease in heart rate rather than SV accounted for most of the decrease in CO we demonstrated.

Owing to the multifactorial aetiology of severe anaemia and that an acute presentation may be followed by a long period of moderate to severe anaemia (chronic) anaemia, our study provides reassuring data for the safety more broadly of blood transfusions. Our data also indicate that the usual parameters that are used to define cardiac overload/failure, including tachycardia, tachypnoea, hepatomegaly and poor perfusion, present in many children in this study (and in the main TRACT), were largely due to compensatory mechanisms rather than a decompensated cardiovascular system. The receipt of a blood transfusion, including children who received whole blood (81%), did not result in adverse outcomes. Indeed, it rapidly normalised the haemodynamic parameters.^13^ The data generated in this substudy should be reassuring, particularly for those concerned that higher volumes may worsen cardiac function. Our data do not support this. Indeed, we showed that higher blood volumes (30 ml/kg whole blood equivalent) resulted in better resolution compared with lower (standard) volume (20 ml/kg whole blood equivalent).

The emergence of non-invasive devices that can be used by non-specialists with minimal training has enabled both clinical and research investigations across diverse settings. In validation studies, accuracy and reliability have been investigated and shown to be as accurate as traditional invasive thermodilution methods,^23, 24^ as well as being more practical and safer. Since its introduction in 2001, the USCOM device has been used in a wide range of clinical settings, including critical care and emergency medicine.^25, 26^ It is a one of the recommended devices to assess haemodynamics for fluid management during resuscitation.^21^ Stroke volume variability (SVV) has been shown to be a reliable parameter to assess fluid responsiveness in adults.^27^ However, in children, a systematic review involving six studies (involving 224 children receiving fluid boluses) suggests it may be of diagnostic relevance, but as there was high heterogeneity, it was concluded that more research was needed to confirm the diagnostic accuracy of SVV in predicting fluid responsiveness in children.^28^

With respect to using surrogate measures such as COI or SVI as parameters to predict better patient-centred outcomes, it is important to interpret the findings of this substudy in the light of the outcome of the TRACT overall. In 1565 children with severe anaemia without clinical signs of severity randomised to either immediate or no immediate transfusion, there was no difference in clinical outcomes at day 28 or at 6 months between the children who received immediate transfusion and the control arm. Outcomes included mortality and Hb recovery. While COM-TRACT demonstrated early improvement in cardiac physiological measurements in the transfused compared with the non-transfused children, by design it assessed evidence of the sustained clinical benefit of immediate transfusion. It serves as another warning to beware of using surrogate physiological measures to infer or predict important clinically relevant outcomes.^29^ Nevertheless, 49% of children randomised in the TRACT to no immediate transfusion ultimately required a transfusion due to a decrease in Hb to <4 g/dl or the development of clinical signs of severity. The median time for transfusion was 24.9 h. These findings, taken together with the findings from this substudy of persistently high heart rate, SVI and COI in non-transfused children, support the need for close haematological and clinical monitoring of non-transfused children, particularly in the first 24 h after admission.

Based on the main TRACT results, a consensus-based guideline was developed for managing African children with severe anaemia,^14^ which, if implemented alongside point-of-care testing,^30^ could result in substantial savings for the blood transfusion services and better outcomes for children. The data from this substudy are reassuring, particularly for those concerned that a higher transfusion volume may worsen cardiac function. Our data do not support this. Resolution of COI and SVI was similar in both the 20 ml/kg and 30 ml/kg groups. Of note, no child received any anti-failure medication, including diuretics, and no child developed evidence of TACO or pulmonary oedema (TRALI).

### Limitations

The major limitation of this study was the relatively small size and the fact that we used a convenience sampling method. A total of 3 of 52 participants were discharged before being discharged from COM-TRACT, hence missing the final USCOM measurement. Considering the findings of the TRACT, a larger study comparing the effect of transfusion volume on myocardial performance of febrile children is warranted.

### Strengths

This physiological study was linked to a well-conducted clinical trial measuring patient-centred clinical outcomes. The receipt of a transfusion and the volume given was determined by randomisation and not clinical preference. The USCOM measurements were made by a clinician independent of the clinical trial team and the data were not shared in real time so as not to affect patient management.

## Conclusions

The data generated by this small but granular study of haemodynamic and cardiac function provide reassuring physiological evidence to show the safety of higher blood transfusion doses (30 ml/kg) than what is currently recommended. It also supports the findings of a secondary analysis of the TRACT indicating that whole blood transfusions are safe. These data support the new evidence-based paediatric transfusion algorithm^22^ for anaemic African children and its recommendation for safe use of whole blood for transfusion without the need for diuretics. We recommend that these data should be reviewed alongside the proposed transfusion guideline and incorporated into national guidelines in Africa.

## Supplementary Material

**Supplementary data**

Supplementary data are available at *International Health* online.

Supplemental File

## Figures and Tables

**Table 1:** Baseline characteristics of children by randomised arm.

Variable	All (N = 52)^a^	No immediate transfusion (n = 26)^b^	20 ml/kg (n = 13)	30 ml/kg (n = 13)	p-Value
Age (months), median (IQR)	39 (25–58)	36.5 (23–45)	39 (25–87)	64 (30–68)	0.07
Male, n (%)	30 (57.7)	14 (53.9)	7(53.9)	9(69.2)	0.06
Weight (kg), median (IQR)	11 (10–15)	11 (10–13)	10 (9–16)	15 (10–20)	0.10
Mid upper arm circumference (cm),	14.0 (13.4–15.2)	14.2 (13.5–15.2)	14.0 (13.3–14.7)	14.0 (12.8–15.5)	0.88
median (IQR)					
Nutrition status, n (%)					
Severe malnutrition	1 (1.9)	1 (3.9)	0 (0)	0 (0)	
Undernutrition	2 (3.9)	0 (0.00)	1 (7.7)	1 (7.7)	0.55
Heart rate (bpm), median (IQR)	143 (127–159)	147 (137–155)	136 (120–166)	127(124–147)	0.12
Severe tachycardia, n (%)^c^	14 (26.9)	7 (26.9)	4 (30.7)	3 (23.1)	0.91
Respiratory rate, median (IQR)	42 (36–54)	46 (36–60)	42 (36–48)	36 (40–48)	0.191
Severe tachypnoea, n (%)^d^	30 (57.7)	18 (69.2)	8 (61.5)	6 (46.1)	0.38
Hepatomegaly present, n (%)^e^	2 (4)	1 (4)	1 (8)	0 (0)	0.60
Temperature (°C), median (IQR)	37.3 (36.5–37.8)	37.6 (36.8–37.8)	37.6 (36.7–38.0)	37.0 (36.5–37.8)	0.71
>37.5°C, n (%)	24 (46.1)	12 (46.1)	7 (53.8)	5 (38.4)	0.73
Shock, n (%)^f^	22 (43.1)	11 (42.3)	6 (46.2)	5 (41.7)	0.97
Hb (g/dl), median (IQR)	5.1 (4.8–5.6)	5.2 (4.8–5.6)	5.1 (5.0–5.6)	5.1 (4.8–5.5)	0.86
Lactate (mmol/l), median (IQR)	3.0 (2.2–4.0)	3.3 (2.6–4.8)	2.7 (1.8–3.0)	3.3 (2.3–4.2)	0.21
>2 mmol/l, n (%)	37 (78.7)	18 (81.1)	8 (66.7)	11 (84.6)	0.82
Malaria positive, n (%)	40 (76.9)	18 (69.2)	10 (76.9)	12 (92.3)	0.27
C-reactive protein (mg/dl), median (IQR)	74 (29–124)	84 (41–125)	47 (13–75)	74 (37–128)	0.31
Previous transfusion, n (%)	5 (9.6)	2 (7.7)	1 (7.7)	2 (15.4)	0.74
HIV positive, n (%)	5 (9.6)	1 (3.9)	2 (15.4)	2 (15.4)	0.22
Sickle cell anaemia, n (%)	3 (5.7)	0 (0.0)	2 (15.4)	1 (7.7)	
Sickle cell Trait (AS)	1 (1.9)	1 (3.9)	0 (0.0)	0 (0.0)	0.31
CO measures					
SV (ml), median (IQR)	27 (22.5–33)	25 (21–30)	26 (22–33)	32 (28–39)	0.03
CO (l/min), median (IQR)	4 (3.3–4.6)	3.9 (2.9–4.2)	3.7 (3.3–4.4)	4.4 (4–4.8)	0.09
SVI (ml/m^2^), median (IQR)	48 (42–54)	47.5 (42–53)	52.0 (43–57)	53.0 (42–56)	0.48
COI (l/min/m^2^), median (IQR)	6.7 (6.1–7.6)	7 (6.1–7.5)	6.7 (6.4–7.4)	6.5 (5.2–9.0)	0.83
Post-admission					
Transfusion with whole blood, %	81	83	92	69	0.32
Required diuretics, n	0	0	0	0	

a Of 52 enrolled, 1 did not have prior blood transfusion information indicated, 1 did not have the shock status information indicated, 5 did not have lactate information indicated and 5 did not have HIV status recorded.

b 6/26 children had a deferred transfusion (5/6 received it >48 h after admission, 1/6 was 26 h after admission).

c Defined as heart rate >180, >160 and >140 bpm for ages <1 y, 1–4 y and ≥ 5 y respectively.

d Defined as >50 and >40 for ages 2–12 months and 1–5 y, respectively.

e Defined as liver >2 cm below the costal margin.

f Defined as any one of the following: capillary refill time >2 s, a lower-limb temperature gradient (a positive temperature gradient was indicated if the peripheral limb was cooler than the thigh) or weak pulse.

## Data Availability

The datasets used and/or analysed during the current study are available from the corresponding author upon reasonable request.
