# Supplementary material for: Cardiac output measurement in Malawian children ages 2 months–12 years hospitalised with severe anaemia (COM-TRACT)
Source: Int Health. 2025 Sep 22;18(4):600–9. doi: 10.1093/inthealth/ihaf103 (PMC7618878; doi:10.1093/inthealth/ihaf103)
Supplement: ihaf103_Supplemental_File [file ihaf103_supplemental_file.docx]

Supplemental Files

**Table S1 Univariate fraction polynomial model regression showing change in SVI at discharge adjusted to 24 hours after randomisation**

| **Unadjusted change in SVI at 24 hours** | | |  |
| --- | --- | --- | --- |
| **Baseline factor** | **Coefficient** | **95% Confidence Interval** | **P value** |
| Transfusion 20ml/kg | -2.00569 | -6.617607 2.606226 | 0.386 |
| **Transfusion 30ml/kg** | **-6.851329** | **-11.60775 -2.094909** | **0.006** |
| **Age** | 0.0272625 | -.0454348 .0999597 | 0.454 |
| **Sex** | -1.315927 | -5.441733 2.809879 | 0.524 |
| **HIV Status** | -0.0988767 | -3.538874 3.341121 | 0.954 |
| **Nutrition Status** | -.680891 | 5.750749 4.388967 | 0.788 |
| **Blood transfusion before** | 2.888261 | -4.720712 10.49723 | 0.449 |
| **Shock** | 2.832479 | -1.238767 6.903724 | 0.168 |
| **Malaria** | 0.2081451 | -4.647327 5.063617 | 0.932 |
| **Sickle cell** | 1.844398 | -4.289729 7.978526 | 0.548 |
| **Haemoglobin** | -0.9947377 | -5.573571 3.584096 | 0.664 |
| **Heart Rate** | .0266898 | -.0770793 .1304588 | 0.607 |
| **Respiratory Rate** | -.0042314 | -.1496957 .141233 | 0.954 |
| **Axillary Temperature** | 1.296784 | -.4773766 3.070944 | 0.148 |

**Table S2. Multivariable fraction polynomial model regression showing change in COI at discharge adjusted to 24 hours after randomisation**

| **Unadjusted COI at 24 hours** | | | | **Adjusted change in COI at 24 hours** | | |
| --- | --- | --- | --- | --- | --- | --- |
| **Baseline factor** | **Coefficient** | **95% Confidence Interval** | **P value** | **Coefficient** | **95% Confidence Interval** | **P value = 0.05** |
| **Transfusion 20ml/kg** | **-1.111775** | **-1.789643**  **-0.4339071** | **0.002** | **-1.10038** | **-1.735448**    **-0.4653123** | **0.001** |
| **Transfusion 30ml/kg** | **-1.686967** | **-2.428762**  **-0.9451712** | **<1** | **-1.656731** | **-2.351992**    **-0.9614703** | **<1** |
| **Age** | 0.0031608 | -0.0092854  0.015607 | 0.612 |  |  |  |
| **Sex** | -0.0053605 | -0.7247954  0.7140744 | 0.988 |  |  |  |
| **HIV Status** | -0.0721009 | -.6634201  0.5192183 | 0.807 |  |  |  |
| **Nutritional Status** | -0.3540955 | -1.255405  0.5472137 | 0.433 |  |  |  |
| **Blood transfusion before** | 0.2379078 | -1.06158  1.537395 | 0.714 |  |  |  |
| **Shock** | 0.6000094 | -0.0853675  1.285386 | 0.085 |  |  |  |
| **Malaria** | -0.7327595 | -1.535144  0.0696248 | 0.072 |  |  |  |
| **Sickle cell** | 0.8869196 | -0.1438362  1.917675 | 0.090 |  |  |  |
| **Haemoglobin** | 0.0903115 | -0.7020528  0.8826758 | 0.820 |  |  |  |
| **Heart Rate** | 0.0066871 | -0.0123005  0.0256747 | 0.482 |  |  |  |
| **Respiratory Rate** | 0.0021511 | -0.0250559 0.0293581 | 0.874 |  |  |  |
| **Axillary Temperature** | **0.3364933** | **0.0424399 0.6305467** | **0.026** | **0.317362** | **0.0813357 0.5533883 (0.010)** |  |
